# Socioeconomic Status and Glycemic Control in Type 2 Diabetes; Race by Gender Differences

**DOI:** 10.3390/healthcare5040083

**Published:** 2017-11-01

**Authors:** Shervin Assari, Maryam Moghani Lankarani, John D. Piette, James E. Aikens

**Affiliations:** 1 Department of Psychiatry, University of Michigan, Ann Arbor, MI 48109, USA; 2 Center for Research on Ethnicity, Culture and Health, School of Public Health, University of Michigan, Ann Arbor, MI 48109, USA; lankaranii@yahoo.com; 3 VA Center for Clinical Management Research, Ann Arbor, MI 48105, USA; jpiette@med.umich.edu; 4 Department of Health Behavior and Health Education, School of Public Health, University of Michigan, Ann Arbor, MI 48109, USA; 5 Department of Family Medicine, University of Michigan, Ann Arbor, MI 48104, USA; aikensj@med.umich.edu

**Keywords:** diabetes, insurance, ethnic groups, glycemic control, African Americans

## Abstract

*Background*: This study aimed to investigate differences in the association between socioeconomic status (SES) and glycemic control in type 2 diabetes mellitus (DM) across race by gender groups. *Methods*: Using a convenient sampling strategy, participants were 112 patients with type 2 DM who were prescribed insulin (ns = 38 Black women, 34 Black men, 14 White women, and 26 White men, respectively). Linear regression was used to test the associations between sociodemographic variables (race, gender, SES, governmental insurance) and Hemoglobin A1c (HbA1c) in the pooled sample and within subgroups defined by race and gender. *Results*: In the pooled sample, neither SES nor governmental insurance were associated with HbA1c. However, the race by gender interaction approached statistical significance (B = 0.34, 95% CI = −0.24–3.00, *p* =0.094), suggesting higher HbA1c in Black women, compared to other race by gender groups. In stratified models, SES (B = −0.33, 95% CI = −0.10–0.00, *p* = 0.050), and governmental insurance (B = 0.35, 95% CI = 0.05–2.42, *p* = 0.042) were associated with HbA1c for Black men, but not for any of the other race by gender subgroups. *Conclusion*: Socioeconomic factors may relate to health outcomes differently across race by gender subgroups. In particular, SES may be uniquely important for glycemic control of Black men. Due to lack of generalizability of the findings, additional research is needed.

## 1. Background

Socioeconomic status (SES) is a major determinant of risk for diabetes (DM) [1,2]. Research from the U.S. [3], Canada [4], and developing countries [5] has suggested that low education [1], low income [3,4,6], being single [5], and minority status [7,8] are all risk factors for DM. Similar trends have been reported for prevalence [3] and incidence [9]. Distribution of DM in the community follows a social gradient, with the highest prevalence in the lowest SES group, and then decline as SES increases [10]. In addition, SES is also a determinant of DM complications and mortality [1]. Among patients with DM, SES is a determinant of QOL [11], depression [12,13], medical comorbidities, medication adherence, and glucose control [14].

Minority status is associated with higher risk [7] and burden of DM in general population [8]. Blacks have a higher risk of DM [8,15], low SES [16,17,18], and worse outcomes associated with DM [15,16,17,18,19,20,21,22,23,24]. Risk factors of DM such as obesity [23], poor nutrition [21], and low exercise [22] are all more common among Blacks, particularly low SES Blacks who live in urban areas. Blacks with DM also suffer worse health outcomes and additional risk of mortality and complications [24]. Compared to Whites with DM, Blacks with DM also receive less frequent care and poorer quality of care [25].

In contrast to the traditional belief that worse health outcomes among minorities is wholly attributable to SES differences, growing evidence suggests that economic and social resources such as SES may affect health outcomes differently across race and gender groups [26]. In this emerging view, instead of explaining (mediating) the effect of race [27,28], SES interacts with race to affect resources and health outcomes.

This study aimed to investigate the association between SES and glycemic control in a clinical sample of White and Black patients with type 2 DM. We were particularly interested in the differential role of SES factors upon glycemic control based on race, gender, and their intersection. In line with previous research in the general population that has shown interactions between SES and race [16,17,18,20], SES by DM [26], and risk factors and race [19,23], we hypothesized that associations between SES factors and glycemic control would vary across subgroups defined by race and gender. This hypothesis is partially based on prior data suggesting that Black patients with DM have worse SES and therefore may have particular sets of needs [29].

## 2. Materials and Methods

### 2.1. Design and Setting

This is a cross-sectional study, in an outpatient setting in a large Midwestern urban health care system. More information regarding methods and design of the study is available elsewhere [30].

### 2.2. Participant Selection

The study used a consecutive sampling strategy. Potential participants were identified using administrative and clinical databases. Eligible patients were mailed a study invitation letter followed by a recruitment telephone call from research staff for further screening and enrollment scheduling.

### 2.3. Eligibility and Sampling

Eligibility criteria included being between 18 and 80 years of age, being able to complete self-report instruments, and having type 2 DM (as indicated by at least one of the following criteria: (1) positive history of hospitalization with a DM-related ICD-9 code (250.x, 357.2, 362.0, or 366.41), (2) two or more outpatient visits with a DM-related ICD-9 code, or (3) prescription for a glucose control medication or monitoring supplies). Type 1 DM was further ruled out by thorough chart review and telephone screening by research staff.

Current analysis only included those who were receiving insulin (*n* = 112). Our sample is not a representative sample, so the results may be affected by selection bias, particularly because not every person who was contacted agreed to participate in the study.

### 2.4. Ethics

The study protocol was approved by institutional review boards at the University of Michigan and the Henry Ford Health System. All participants provided informed consent. Data were kept confidential.

### 2.5. Measures

Main outcome: Glycemic control (Hemoglobin A1c; HbA1c) was measured with the DCA 2000 (GMI, Ramsey, MN, USA), which analyzes capillary blood samples through a monoclonal antibody method [30].

Socioeconomic status (SES) and governmental insurance: We measured SES using the U.S. Census Bureau Index of Socioeconomic Status [31], adjusted for the regional Consumer Price Index at the time of survey [30]. A higher score was indicative of better SES. Governmental insurance in this study was Medicaid coverage [32].

Race: Participants were asked to classify themselves using U.S. Census racial/ethnic categories. Race included White (Caucasian) or Black (African American). 

Demographic Factors: The study collected data on self-reported age and gender. Age was treated as a continuous measure, and gender was female versus male [reference category].

Clinical Variables: Diabetes duration was our main clinical variable that was conceptualized as a covariate. Diabetes duration was calculated as number of years passed since diagnosis, and was self-reported. Diabetes duration was treated as a continuous measure in this study. 

### 2.6. Analysis

Data were analyzed using Stata 13.0 (Stata Corp., College Station, TX, USA) as well as Statistical Package for Social Sciences v.20 (SPSS 20). Descriptive statistics such as mean (SD) and frequency (%) were used to describe the variables in the pooled sample as well as race by gender subgroups. For bivariate analysis, we tested Pearson correlations between study variables across groups based on the intersection of race and gender. We used linear regression models to estimate adjusted associations between race, SES, and HbA1c, net of demographics. The outcome variable was HbA1c, and covariates were age and gender. Model 1 in the pooled sample only had main effects. Model 2 in the pooled sample also included the interaction between race and gender. Finally, we ran four stratified models for Black and White men and women. Standardized B, 95% CI, and *p*-values were reported. A *p*-value of <0.05 was considered significant. 

## 3. Results

The current analysis included 112 DM patients, which was composed of 38 Black women, 34 Black men, 14 White women, and 26 White men. Table 1 describes the descriptive statistics in the pooled sample and subgroups based on the race by gender (Table 1).

Table 2 shows correlation coefficients between study variables in the pooled sample and based on race by gender subgroups. SES was marginally correlated with HbA1c in Black men. SES was not correlated with HbA1c in Black women, White men, and White women. Age and governmental insurance were not correlated among Black men, although they were correlated among White men, White women, and Black women. 

As shown in Table 3, in the absence of race by gender interaction term, age (B = −0.25, 95% CI = −0.11–0.00, *p* = 0.046) was associated with HbA1c in the pooled sample; gender was possibly associated with HbA1c (B = −0.19, 95% CI = −1.47–0.04, *p* = 0.064); and race, SES, DM duration, and governmental insurance were not associated with HbA1c. Race by gender interaction term was marginally significant on glycemic control (B = 0.34, 95% CI = −0.24–3.00, *p* = 0.094). 

As shown in Table 4, age, SES, governmental insurance, and DM duration were not associated with HbA1c in White men, White women, and Black women. In Black men, however, age (B = −0.35, 95% CI = −0.20–0.01, *p* = 0.040), and governmental insurance (B = 0.35, 95% CI = 0.05–2.42, *p* = 0.042) were each significantly associated with HbA1c, and SES (B = −0.33, 95% CI = −0.10–0.00, *p* = 0.050) was possibly associated with HbA1c.

## 4. Discussion

In racially diverse samples of insulin-treated patients with type 2 DM, data analyses revealed two findings: First, we found a marginally significant interaction between race and gender on glycemic control, suggesting worse glycemic control in Black women compared to Black men and White women. Second, although SES and governmental insurance were not associated with HbA1c in the pooled sample, race by gender difference were found in these effects. SES and governmental insurance emerged as a predictor of glycemic control for Black men but not White men, White women, or Black women. 

Regarding our first finding, worse glycemic control of Black women may be due to their use of over-eating and binge eating in response to stress [33,34]. This finding suggests a more aggressive DM control for Black women who may have worse glycemic control compared to other race by gender groups. 

Our second finding that SES resources may differently affect sub-populations is in line with previous research that has shown that the same increase in SES generates various levels of change in health outcomes of race, gender, and SES groups [26]. Our finding refutes the traditional assumption that SES is universally protective against undesired health outcomes of all social and demographic groups. Recent research has shown that race and gender do not only alter exposure to risk and protective factors [27,28] but also alter vulnerability to them [20,35,36,37,38,39,40,41]. We also found that Medicaid coverage was associated with worse glycemic control of Black men, which may be due to higher health need of individuals who qualify for Medicaid coverage. So, we believe we attribute this finding to selection rather than causation.

Racial minority status alters the health gains that are expected to follow SES [35]. In a study among the elderly, race interacted with education level on alcohol consumption, with education having a smaller effect on drinking patterns for Blacks than it does for Whites. While among Whites, high-school graduation and college graduation were both associated with increased odds of ever drinking, among Blacks, high-school graduation, but not college graduation, was associated with ever drinking [35]. Other research has shown that education [37] and employment [20] have larger protective effects against premature mortality for Whites than Blacks. The same is true for neighborhood quality [42]. However, our present findings contradict these results, by showing effect of SES for Black men but not for any other groups. 

SES effects depend upon race [35] and gender [36,38,40,43,44,45,46]. A wide range of social environment may differentially affect metabolic risk across social groups [36,38,40,43,44,45,46]. Contradicting our findings, however, most previous research has documented a more salient role of social and psychological constructs on metabolic risk of females than males [36,38,40,43,44,45,46]. 

Our findings are in line with the studies showing that SES may have a larger effect on the health of men than women [47,48]. Specifically, men may be more susceptible to the effects of unemployment than women [47,48,49]. Larger health gain of men from employment was confirmed by a meta-analysis [50]. However, not every study has shown larger effects of SES for males than females [51,52].

Not only race and gender—but their also intersection—shape how SES indicators affect health [27,28,53,54]. For example, in a recent study, the protective effects of education and income on sustained insomnia, physical inactivity, and BMI varied across race by gender groups. Education did not protect Black men against insomnia, physical inactivity, and BMI. Education did not protect Black women against high BMI. Income had a protective effect against BMI among White and Black women but not White and Black men [43]. Nonetheless, intersections of social identities alter the health consequences of social risk and protective factors [52].

More research is needed on how race, gender, and SES impact health of individuals with DM. While this study in clinical sample showed a larger effect of SES on health among Black men, most previous research on the community sample has shown smaller effects of SES on health for Blacks than Whites [20,37,39]. Our findings did not support the “Blacks’ diminished return hypothesis” [55].

In our sample, Black males and females developed diabetes in a younger age compared to White men and women. This pattern is in line with the lower age of onset of chronic disease in minorities particularly Blacks [56]. Blacks and other minority populations have higher prevalence of CMC [57,58]. Compared with Whites, Black Americans have greater physical health morbidity and mortality at every age [59]. In several studies, chronic diseases are shown to fully mediate the lower life expectancy of Blacks compared to Whites [60,61]. Overall, chronic disease are more common and more consequential for Blacks than Whites [54].

Our findings may have clinical and policy implications. Similar to research on other health conditions [62,63,64], we recommend that DM interventions should be tailored by race and gender [63,65,66]. This is especially important as minority populations may be more receptive to programs and interventions that are tailored to their specific needs. As loss of insurance may have the largest negative effects on glycemic control of Black men. Policies that promote universal access of all populations to a new resource such as insurance may result in differential health gain across diverse populations [20,35,36,37,38,39,40,41]. Optimal solutions should go beyond universal interventions by considering specific health needs of each demographic group [67]. 

To conclude, the effects of SES on glycemic control vary across the race by gender subgroups of insulin-treated type 2 DM patients. Specifically, SES may be particularly important for glycemic control of Black men. Further research with a large and diverse sample is needed given the degree of inconsistency across the literature. 

## 5. Conclusions

Socioeconomic factors have differential effects on health outcomes across race by gender subgroups. In particular, SES may have a larger effect on glycemic control of Black men with DM. Due to lack of generalizability of the findings, additional research is needed.

## Figures and Tables

**Table 1:** Descriptive statistics for pooled sample and race by gender subgroups.

	Pooled Sample *n* = 112		White Men *n* = 26		White Women *n* = 14		Black Men *n* = 34		Black Women *n* = 38	
	Mean	SD	Mean	SD	Mean	SD	Mean	SD	Mean	SD
Age (years)	55.79	8.94	61.46	9.84	60.36	8.34	50.68	7.88	54.82	6.27
SES	66.23	16.66	66.19	15.35	62.27	13.66	69.22	14.27	65.01	20.36
Diabetes duration (years)	13.48	8.84	18.33	13.09	13.82	5.86	12.68	7.00	10.74	6.10
HbA1c	8.02	1.87	7.86	1.26	7.09	1.59	8.63	2.22	7.93	1.84

SES: Socioeconomic Status; HbA1c: Hemoglobin A1c.

**Table 2:** Pearson correlations for the pooled sample and race by gender subgroups.

	1	2	3	4	5
**Pooled Sample**					
1 Age	---	−0.13	0.47 **	0.39 **	−0.32 **
2 SES		---	−0.27 **	−0.18 ^#^	0.02
3 Governmental insurance			---	0.01	−0.23 *
4 Diabetes duration				---	−0.08
5 HbA1c					---
**White Men**					
1 Age	---	−0.28	0.71 **	0.28	−0.39 *
2 SES		---	−0.25	−0.16	0.04
3 Governmental insurance			---	−0.10	−0.29
4 Diabetes duration				---	−0.12
5 HbA1c					---
**White Women**					
1 Age	---	−0.16	0.79 **	0.47	0.04
2 SES		---	−0.48 ^#^	0.54 ^#^	−0.27
3 Governmental insurance			---	0.28	0.02
4 Diabetes duration				---	−0.26
5 HbA1c					---
**Black Men**					
1 Age	---	0.01	−0.04	0.36 *	−0.35 *
2 SES		---	−0.12	−0.24	−0.30 ^#^
3 Governmental insurance			---	−0.26	−0.27
4 Diabetes duration				---	−0.01
5 HbA1c					---
**Black Women**					
1 Age	---	−0.03	0.34 *	0.35 *	−0.23
2 SES		---	−0.29 ^#^	−0.36 *	0.24
3 Governmental insurance			---	0.10	−0.18
4 Diabetes duration				---	−0.06
5 HbA1c					---

SES: Socioeconomic Status, HbA1c: Hemoglobin A1c. ^#^: 0.05 ≤ *p* ≤ 0.10 (2-tailed). *: 0.01 < *p* < 0.05 (2-tailed). **: *p* ≤ 0.01 (2-tailed).

**Table 3:** Summary of linear regression in the pooled sample.

Variable	Model 1			Model 2		
	B	95% CI	*p*	B	95% CI	*p*
Race (Black)	0.12	−0.39–1.38	0.267	−0.02	−1.17–1.03	0.898
Gender (female)	−0.19	−1.47–0.04	0.064	−0.44	−3.00–0.32	0.016
Age (years)	−0.25	−0.11–0.00	0.046	−0.29	−0.12–0.01	0.022
SES	−0.03	−0.03–0.02	0.727	−0.03	−0.03–0.02	0.790
Governmental insurance	0.11	−0.28–0.82	0.326	0.10	−0.29–0.80	0.360
Diabetes duration (years)	0.00	−0.05–0.05	0.994	0.00	−0.05–0.05	0.973
Race × Gender (Black Women)	-	-	-	0.34	−0.24–3.00	0.094

SES: Socioeconomic Status. Outcome is Hemoglobin A1c (HbA1c).

**Table 4:** Summary of linear regression stratified by race by gender groups.

	White Men			White Women			Black Men			Black Women		
	B	95% CI	*p*	B	95% CI	*p*	B	95% CI	*p*	B	95% CI	*p*
Age (years)	−0.46	−0.15–0.04	0.214	0.18	−0.24–0.31	0.762	−0.35	−0.20–0.01	0.040	−0.25	−0.20–0.05	0.217
SES	−0.03	−0.04–0.04	0.889	−0.13	−0.12–0.10	0.847	−0.33	−0.10–0.00	0.050	0.27	−0.01–0.06	0.176
Governmental insurance	−0.06	−1.03–0.88	0.868	0.52	−1.38–2.55	0.482	0.35	0.05–2.42	0.042	0.02	−1.12–1.27	0.902
Diabetes duration (years)	0.00	−0.05–0.05	0.984	0.03	−0.28–0.29	0.961	−0.08	−0.16–0.10	0.634	0.13	−0.09–0.17	0.544

SES: Socioeconomic Status. Outcome is Hemoglobin A1c (HbA1c).

## References

[1] Saydah S., Lochner K. (2010). Socioeconomic status and risk of diabetes-related mortality in the U.S.. Public Health Rep..

[2] Rabi D.M., Edwards A.L., Southern D.A., Svenson L.W., Sargious P.M., Norton P., Larsen E.T., Ghali W.A. (2006). Association of socio-economic status with diabetes prevalence and utilization of diabetes care services. BMC Health Serv. Res..

[3] Dyck R., Karunanayake C., Pahwa P., Hagel L., Lawson J., Rennie D., Dosman J., Saskatchewan Rural Health Study Group (2013). Prevalence, risk factors and co-morbidities of diabetes among adults in rural Saskatchewan: The influence of farm residence and agriculture-related exposures. BMC Public Health.

[4] Lee D.S., Chiu M., Manuel D.G., Tu K., Wang X., Austin P.C., Mattern M.Y., Mitiku T.F., Svenson L.W., Putnam W. (2009). Trends in risk factors for cardiovascular disease in Canada: Temporal, socio-demographic and geographic factors. CMAJ.

[5] Tripathy J.P., Thakur J.S., Jeet G., Chawla S., Jain S., Pal A., Prasad R., Saran R. (2017). Prevalence and risk factors of diabetes in a large community-based study in North India: Results from a STEPS survey in Punjab, India. Diabetol. Metab. Syndr..

[6] Bird Y., Lemstra M., Rogers M., Moraros J. (2015). The relationship between socioeconomic status/income and prevalence of diabetes and associated conditions: A cross-sectional population-based study in Saskatchewan, Canada. Int. J. Equity Health.

[7] Carter J.S., Pugh J.A., Monterrosa A. (1996). Non-insulin-dependent diabetes mellitus in minorities in the United States. Ann. Intern. Med..

[8] Tull E.S., Roseman J.M. (1995). Diabetes in African Americans. Diabetes in America.

[9] Lysy Z., Booth G.L., Shah B.R., Austin P.C., Luo J., Lipscombe L.L. (2013). The impact of income on the incidence of diabetes: A population-based study. Diabetes Res. Clin. Pract..

[10] Lipscombe L.L., Austin P.C., Manuel D.G., Shah B.R., Hux J.E., Booth G.L. (2010). Income-related differences in mortality among people with diabetes mellitus. CMAJ.

[11] Glasgow R.E., Ruggiero L., Eakin E.G., Dryfoos J., Chobanian L. (1997). Quality of life and associated characteristics in a large national sample of adults with diabetes. Diabetes Care.

[12] Tellez-Zenteno J.F., Cardiel M.H. (2002). Risk factors associated with depression in patients with type 2 diabetes mellitus. Arch. Med. Res..

[13] Mier N., Bocanegra-Alonso A., Zhan D., Wang S., Stoltz S.M., Acosta-Gonzalez R.I., Zuniga M.A. (2008). Clinical depressive symptoms and diabetes in a binational border population. J. Am. Board Fam. Med..

[14] Houle J., Lauzier-Jobin F., Beaulieu M.D., Meunier S., Coulombe S., Côté J., Lespérance F., Chiasson J.L., Bherer L., Lambert J. (2016). Socioeconomic status and glycemic control in adult patients with type 2 diabetes: A mediation analysis. BMJ Open Diabetes Res. Care.

[15] Marshall M. (2004). Diabetes in African Americans. Postgrad. Med. J..

[16] Williams D.R. (1999). Race, socioeconomic status, and health the added effects of racism and discrimination. Ann. N. Y. Acad. Sci..

[17] Ren X.S., Amick B.C., Williams D.R. (1998). Racial/ethnic disparities in health: The interplay between discrimination and socioeconomic status. Ethnicity Dis..

[18] Williams D.R., Mohammed S.A., Leavell J., Collins C. (2010). Race, socioeconomic status, and health: Complexities, ongoing challenges, and research opportunities. Ann. N. Y. Acad. Sci..

[19] Conway B.N., May M.E., Blot W.J. (2012). Mortality among low-income African Americans and whites with diabetes. Diabetes Care.

[20] Assari S., Lankarani M.M. (2016). Race and Urbanity Alter the Protective Effect of Education but not Income on Mortality. Front. Public Health..

[21] Satia J.A. (2009). Diet-related disparities: Understanding the problem and accelerating solutions. J. Am. Diet. Assoc..

[22] Winkleby M.A., Kraemer H.C., Ahn D.K., Varady A.N. (1998). Ethnic and Socioeconomic differences in cardiovascular disease risk factors findings for women from the Third National Health and Nutrition Examination Survey, 1988–1994. JAMA.

[23] Zhang Q., Wang Y., Huang E.S. (2009). Changes in racial/ethnic disparities in the prevalence of Type 2 diabetes by obesity level among US adults. Ethnicity Health.

[24] Cowie C.C., Port F.K., Rust K.F., Harris M.I. (1994). Differences in survival between black and white patients with diabetic endstage renal disease. Diabetes Care.

[25] Harris M.I. (1990). Noninsulin-dependent diabetes mellitus in black and white Americans. Diabetes Metab. Rev..

[26] Moghani Lankarani M., Assari S. (2017). Diabetes, Hypertension, Obesity and Long Term Risk of Renal Disease Mortality; Racial and Socioeconomic Differences. J. Diabetes Investig..

[27] Assari S. (2016). Distal, intermediate, and proximal mediators of racial disparities in renal disease mortality in the United States. J. Nephropathol..

[28] Assari S. (2017). Number of Chronic Medical Conditions Fully Mediates the Effects of Race on Mortality; 25-Year Follow-Up of a Nationally Representative Sample of Americans. J. Racial Ethnic Health Disparities.

[29] Batts M.L., Gary T.L., Huss K., Hill M.N., Bone L., Brancati F.L. (2001). Patient priorities and needs for diabetes care among urban African Americans adults. Diabetes Educ..

[30] Wagner J.A., Perkins D.W., Piette J.D., Lipton B., Aikens J.E. (2009). Racial differences in the discussion and treatment of depressive symptoms accompanying type 2 diabetes. Diabetes Res. Clin. Pract..

[31] Assari S. (2017). Life Expectancy Gain Due to Employment Status Depends on Race, Gender, Education, and Their Intersections. J. Racial Ethnic Health Disparities.

[32] Doll K.M., Basch E.M., Meng K., Barber E.L., Gehrig P.A., Brewster W.R., Meyer A.M. (2016). Clinical Benefits Associated with Medicaid Coverage Before Diagnosis of Gynecologic Cancers. J. Oncol. Pract..

[33] Bowleg L. (2012). The problem with the phrase women and minorities: Intersectionality-an important theoretical framework for public health. Am. J. Public Health.

[34] Assari S., Lee D.B., Nicklett E.J., Moghani Lankarani M., Piette J.D., Aikens J.E. (2017). Racial Discrimination in Health Care Is Associated with Worse Glycemic Control among Black Men but Not Black Women with Type 2 Diabetes. Front. Public Health.

[35] Blostein F., Assari S., Caldwell C.H. (2017). Gender and Ethnic Differences in the Association between Body Image Dissatisfaction and Binge Eating Disorder among Blacks. J. Racial Ethnic Health Disparities.

[36] Assari S., Caldwell C.H. (2017). Neighborhood Safety and Major Depressive Disorder in a National Sample of Black Youth; Gender by Ethnic Differences. Children.

[37] Assari S., Nikahd A., Malekahmadi M.R., Lankarani M.M., Zamanian H. (2017). Race by Gender Group Differences in the Protective Effects of Socioeconomic Factors against Sustained Health Problems across Five Domains. J. Racial Ethnic Health Disparities.

[38] Assari S., Moghani Lankarani M., Caldwell C.H., Zimmerman M.A. (2016). Fear of Neighborhood Violence during Adolescence Predicts Development of Obesity a Decade Later: Gender Differences among African Americans. Arch. Trauma Res..

[39] Assari S., Moghani Lankarani M., Kazemi Saleh D., Ahmadi K. (2013). Gender modifies the effects of education and income on sleep quality of the patients with coronary artery disease. Int. Cardiovasc. Res. J..

[40] Lankarani M.M., Shah S., Assari S. (2017). Gender Differences in Vulnerability to Socioeconomic Status on Self-Rated Health in 15 Countries. Womens Health Bull..

[41] Carter J.D., Assari S. (2017). Sustained Obesity and Depressive Symptoms over 6 Years: Race by Gender Differences in the Health and Retirement Study. Front. Aging Neurosci..

[42] Garcy A.M., Vågerö D. (2012). The length of unemployment predicts mortality, differently in men and women, and by cause of death: A six year mortality follow-up of the Swedish 1992–1996 recession. Soc. Sci. Med..

[43] Assari S. (2016). Psychosocial Correlates of Body Mass Index in the United States: Intersection of Race, Gender and Age. Iran. J. Psychiatry Behav. Sci..

[44] Assari S., Caldwell C.H., Zimmerman M.A. (2015). Low parental support in late adolescence predicts obesity in young adulthood; Gender differences in a 12-year cohort of African Americans. J. Diabetes Metab. Disord..

[45] Assari S. (2017). Perceived Neighborhood Safety Better Predicts Risk of Mortality for Whites than Blacks. J. Racial Ethnic Health Disparities.

[46] Mustard C.A., Bielecky A., Etches J., Wilkins R., Tjepkema M., Amick B.C., Smith P.M., Aronson K.J. (2013). Mortality following unemployment in Canada, 1991–2001. BMC Public Health.

[47] Tsai S.L., Lan C.F., Lee C.H., Huang N., Chou Y.J. (2004). Involuntary unemployment and mortality in Taiwan. J. Formos Med. Assoc..

[48] Roelfs D.J., Shor E., Davidson K.W., Schwartz J.E. (2011). Losing life and livelihood: A systematic review and meta-analysis of unemployment and all-cause mortality. Soc. Sci. Med..

[49] Bauermeister J.A., Pingel E.S., Jadwin-Cakmak L., Harper G.W., Horvath K., Weiss G., Dittus P. (2015). Acceptability and preliminary efficacy of a tailored online HIV/STI testing intervention for young men who have sex with men: The Get Connected! program. AIDS Behav..

[50] Mustanski B., Garofalo R., Monahan C., Gratzer B., Andrews R. (2013). Feasibility, acceptability, and preliminary efficacy of an online HIV prevention program for diverse young men who have sex with men: The keep it up! intervention. AIDS Behav..

[51] Assari S., Caldwell C.H. (2017). Low Family Support and Risk of Obesity among Black Youth: Role of Gender and Ethnicity. Children.

[52] Assari S., Lankarani M.M., Piette J.D., Aikens J.E. (2017). Self-Rated Health and Glycemic Control in Type 2 Diabetes: Race by Gender Differences. J. Racial Ethnic Health Disparities.

[53] Striegel-Moore R.H., Wilfley D.E., Pike K.M., Dohm F.A., Fairburn C.G. (2000). Recurrent binge eating in black American women. Arch. Fam. Med..

[54] Jackson J.S., Knight K.M., Rafferty J.A. (2010). Race and unhealthy behaviors: Chronic stress, the HPA axis, and physical and mental health disparities over the life course. Am. J. Public Health.

[55] Williams I.C., Utz S.W., Hinton I., Yan G., Jones R., Reid K. (2014). Enhancing diabetes self-care among rural African Americans with diabetes: Results of a two-year culturally tailored intervention. Diabetes Educ..

[56] Finkelstein A.N., Taubman S.L., Allen H.L., Wright B.J., Baicker K. (2016). Effect of Medicaid Coverage on ED Use—Further Evidence from Oregon’s Experiment. N. Engl. J. Med..

[57] Jackson J.S., Kastenbaum R. (1993). African American experiences through the adult years. The Encyclopedia of Adult Development.

[58] Hoffman C., Rice D., Sung H.Y. (1996). Persons with chronic conditions: Their prevalence and costs. JAMA.

[59] Mark T.L., Lubran R., McCance-Katz E.F., Chalk M., Richardson J. (2015). Medicaid coverage of medications to treat alcohol and opioid dependence. J. Subst. Abuse Treat..

[60] Watkins D.C., Assari S., Johnson-Lawrence V. (2015). Race and Ethnic Group Differences in Comorbid Major Depressive Disorder, Generalized Anxiety Disorder, and Chronic Medical Conditions. J. Racial Ethnic Health Disparities.

[61] Assari S., Zivin K., Burgard S. (2015). Long-term reciprocal associations between depressive symptoms and number of chronic medical conditions: Longitudinal support for black? White Health Paradox. J. Racial Ethnic Health Disparities.

[62] Sawyer A.M., King T.S., Weaver T.E., Sawyer D.A., Varrasse M., Franks J., Watach A., Kolanowski A.M., Richards K.C. (2017). A Tailored Intervention for PAP Adherence: The SCIP-PA Trial. Behav. Sleep Med..

[63] Farmer M.M., Ferraro K.F. (2005). Are racial disparities in health conditional on socioeconomic status?. Soc. Sci. Med..

[64] Utz S.W., Williams I.C., Jones R., Hinton I., Alexander G., Yan G., Moore C., Blankenship J., Steeves R., Oliver M.N. (2008). Culturally tailored intervention for rural African Americans with type 2 diabetes. Diabetes Educ..

[65] Collins-McNeil J., Edwards C.L., Batch B.C., Benbow D., McDougald C.S., Sharpe D. (2012). A culturally targeted self-management program for African Americans with type 2 diabetes mellitus. Can. J. Nurs. Res..

[66] Assari S. (2017). Combined racial and gender differences in the long-term predictive role of education on depressive symptoms and chronic medical conditions. J. Racial Ethnic Health Disparities.

[67] Assari S. (2017). Unequal gain of equal resources across racial groups. Int. J. Health Policy Manag..

